# Diosgenin and 5-Methoxypsoralen Ameliorate Insulin Resistance through ER-*α*/PI3K/Akt-Signaling Pathways in HepG2 Cells

**DOI:** 10.1155/2016/7493694

**Published:** 2016-08-31

**Authors:** Ke Fang, Hui Dong, Shujun Jiang, Fen Li, Dingkun Wang, Desen Yang, Jing Gong, Wenya Huang, Fuer Lu

**Affiliations:** ^1^Institute of Integrated Traditional Chinese and Western Medicine, Tongji Hospital, Tongji Medical College, Huazhong University of Science and Technology, Wuhan, Hubei 430030, China; ^2^College of Chemistry and Chemical Engineering, Huazhong University of Science and Technology, Wuhan, Hubei 430074, China; ^3^Department of Pharmacology, Hubei University of Chinese Medicine, Wuhan, Hubei 430065, China

## Abstract

To determine the effects and the underlying mechanism of diosgenin (DSG) and 5-methoxypsoralen (5-MOP), two main active components in the classical Chinese prescription Hu-Lu-Ba-Wan (HLBW), on insulin resistance, HepG2 cells were incubated in medium containing insulin. Treatments with DSG, 5-MOP, and their combination were performed, respectively. The result showed that the incubation of HepG2 cells with high concentration insulin markedly decreased glucose consumption and glycogen synthesis. However, treatment with DSG, 5-MOP, or their combination significantly reversed the condition and increased the phosphorylated expression of estrogen receptor-*α* (ER*α*), sarcoma (Src), Akt/protein kinase B, glycogen synthase kinase-3*β* (GSK-3*β*), and the p85 regulatory subunit of phosphatidylinositol 3-kinase p85 (PI3Kp85). At the transcriptional level, expression of the genes mentioned above also increased except for the negative regulation of GSK-3*β* mRNA. The increased expression of glucose transport-4 (GLUT-4) was meanwhile observed through immunofluorescence. Nevertheless, the synergistic effect of DSG and 5-MOP on improving glycometabolism was not obvious in the present study. These results suggested that DSG and 5-MOP may improve insulin resistance through an ER-mediated PI3K/Akt activation pathway which may be a new strategy for type 2 diabetes mellitus, especially for women in an estrogen-deficient condition.

## 1. Introduction

The prevalence of diabetes mellitus is increasing year by year around the world. Not only does it reduce people's quality of life, but also it leads to death for its series of complications. According to an estimate by the International Diabetes Federation, the global prevalence of diabetes mellitus among adults with 20–79 years of age was 8.3% in 2013 [1]. In the Southeast Asian region, 19.1% of all-cause mortality in 50–59-year-old men and 25.7% of all-cause mortality in 50–59-year-old women were attributable to diabetes. Menopausal women seemed to have a striking increase in the incidence of type 2 diabetes (T2DM) [2]. A large portion of healthy postmenopausal women showed decreased insulin sensitivity [3]. Therefore, we speculated that estrogen may play a vital role in maintaining glucose homeostasis. Despite abundant evidence of the validity of estrogen-containing therapies on alleviating menopausal symptoms [4], many women resorted to herbal medicines to regulate blood glucose because of side effects.

In China, herbal medicines have been used in the treatment of diabetes for thousands of years. Hu-Lu-Ba-Wan (HLBW) is a significant formula in traditional Chinese medicine described in the book* Yang Shi Jia Cang Fang*. This prescription consisting of* Trigonella foenum-graecum* and* Psoralea corylifolia* was mainly applied to ameliorate sexual dysfunction in the past. In clinic, we have found HLBW to have a positive effect on blood glucose in patients suffering from T2DM. This hypoglycemic effect has also been proved by our previous study on type 2 diabetic rats [5].

**Figure 1:**
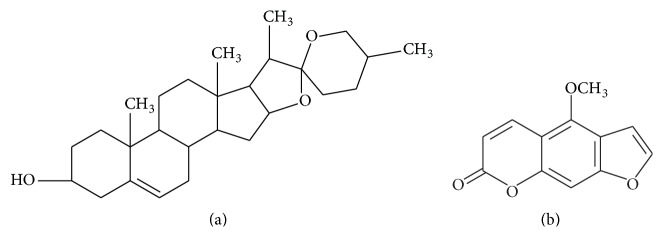
Chemical structure of (a) DSG and (b) 5-MOP.

DSG (Figure 1(a)) is an important precursor of steroidal hormones and can be found in* Trigonella foenum-graecum*. Many studies have reported the beneficial effect of DSG on the treatment of diabetes [6, 7]. 5-MOP (Figure 1(b)) is a kind of phytoestrogen that can be extracted from* Psoralea corylifolia*. Its hypoglycemic effect has also been discovered in vivo and in vitro [8, 9]. Though their estrogen-like effects have been reported [10, 11], relatively little research has examined their direct effect on glycometabolism through the estrogen receptor, which is distributed in multiple organs and mediates estrogen action.

In the human body, glucose is metabolized in specific target organs such as the liver where synthesis and breakdown of hepatic glycogen take place. Under the circumstance of insulin resistance, there is a deficiency in liver glucose uptake and glycogen synthesis, which in turn causes elevated plasma glucose. As a result, decreased insulin sensitivity eventually leads to the occurrence of T2DM. The PI3K/Akt-signaling pathway is the main downstream molecular pathway of insulin and plays an essential role in mobilizing glucose through promoting the expression and translocation of GLUT-4 [12, 13]. Therefore, the objective of this study is to determine whether there is crosstalk between the ER-mediated hypoglycemic effect of the two phytoestrogens and the classical PI3K/Akt-signaling pathway in HepG2 cells.

## 2. Materials and Methods

### 2.1. Chemicals and Reagents

Bovine serum albumin was purchased from Biological Industries Israel Beit Haemek Ltd. (Israel). Roswell Park Memorial Institute-1640 (RPMI-1640) was purchased from Hyclone Laboratories Inc. (Logan, UT, USA). DSG was obtained from Aoke Biology Research Co., Ltd. (Beijing, China). *β*-Estradiol was purchased from Aladdin Industrial Corporation (Shanghai, China). 5-MOP, human insulin, dimethyl sulfoxide (DMSO), and wheat germ agglutinin (WGA) dye were purchased from Sigma-Aldrich Co. (St. Louis, MO, USA). Trypsin, penicillin, streptomycin, 3-(4,5-dimethylthiazol-2-yl)-2,5-diphenyltetrazolium bromide (MTT) assay kits, Western blot kit, 4′,6-diamidino-2-phenylindole (DAPI), Triton X-100, and antifade mounting medium were purchased from Guge Biological Technology Co. (Wuhan, China). Bicinchoninic acid (BCA) protein assay kit was obtained from Biosci Biotechnology Co., Ltd. (Wuhan, Hubei, China). Glucose assay kit was purchased from Beijing Applygen Technologies Inc. (Beijing, China). Hepatic glycogen assay kit was purchased from Nanjing Jiancheng Bioengineering Institute (Nanjing, China). Monoclonal antibody against PI3Kp85, ER*α*, and GLUT-4 were purchased from Millipore Corporation (Billerica, MA, USA). Monoclonal antibody against Akt, p-Akt (Ser473), GSK-3*β*, p-GSK-3*β* (Ser9), Src, and p-Src (Tyr-416) were purchased from Cell Signaling Technology (Beverly, MA, USA). Polyclonal antibody against p-ER*α* (Tyr-537) was purchased from Santa Cruz Biotechnology (Santa Cruz, CA, USA). Trizol reagent, PrimeScript RT reagent kit, and SYBR Premix Ex Taq were purchased from TaKaRa Bio Inc. (Dalian, Liaoning, China). Fluorescent-marked second antibody was provided by LI-COR Biosciences (Lincoln, NE, USA). Stripping buffer was obtained from Beyotime Institute of Biotechnology (Shanghai, China). Dylight 488 (Goat Anti-Rabbit IgG) and Dylight 549 (Goat Anti-Mouse IgG) were purchased from Abbkine, Inc. (Redlands, CA, USA).

### 2.2. Cell Culture and Treatment

HepG2 cells, provided by the Department of Immunology, Tongji Medical College, Huazhong University of Science and Technology, were cultured in RPMI-1640 medium supplemented with 10% FBS, 100 units/mL penicillin, and 100 *μ*g/mL streptomycin and maintained at 37°C in a humidified atmosphere of 5% CO_2_ and 95% air. To ensure cell viability during prolonged incubation of DSG, 5-MOP, and *β*-Estradiol in modified RMPI 1640 medium, cell viability was evaluated by MTT assay according to the manufacturer's protocol.

Approximately 3 × 10^5^ cells/well were transferred into 6-well plates and allowed to grow overnight to 70% confluence. After 10–12 h starvation in RPMI-1640 medium without FBS, media in model and intervention groups were replaced by RPMI-1640 medium containing insulin (10^−6 ^mol/L) for 36 h. For different intervention groups, the medium containing DSG, 5-MOP, DSG + 5-MOP, or *β*-Estradiol was then added, respectively.

### 2.3. Measurement of Glucose Content in Cell Supernatant and Intracellular Glycogen Content

After 24 h drug stimulation, the supernatant was collected to determine the glucose consumption by a glucose assay kit using the glucose oxidase method. Cells were then rinsed twice with phosphate-buffered saline (PBS). Cell suspension was obtained by trypsin digestion and then centrifuged and resuspended with normal saline three times. Cells were broken by supersonic technique (VCX150, Sonics & Materials, Newton, CT, USA). Intracellular glycogen content was measured by a hepatic glycogen assay kit using the sulfuric acid anthrone colorimetric method.

### 2.4. Western Blot Analysis

HepG2 cells were washed with PBS twice and lysed at 4°C with radioimmunoprecipitation assay (RIPA) buffer containing phenylmethanesulfonyl fluoride (PMSF) and protease inhibitor cocktail. Cell debris was removed by centrifugation at 12,000 ×g for 15 min at 4°C, and the supernatant was assayed for protein concentration using the BCA method. Fifty *μ*g protein was solubilized in SDS loading buffer and heated in boiling water for 10 min; then it was separated on 10% SDS-PAGE (120 v, 90 min) and transferred onto nitrocellulose (NC) membranes (280 mA, 90 min). The membranes then were blocked with 5% BSA powder in ultrapure water for 1 h, followed by incubation with primary antibodies (p-ER*α*, ER*α*, p-Akt, Akt, p-Src, Src, p-GSK-3*β*, GSK-3*β*, PI3Kp85, and *β*-actin) overnight at 4°C. The membranes were washed TBST three times for 5 min each and incubated with fluorescence-labeled secondary antibodies for 1 h at room temperature. Then the membranes were washed four times in TBST for 5 min each. For detection, the bands were visualized using a near-infrared fluorescence imaging system (Odyssey, Lincoln, NB, USA). Band densities were quantified by Image-Pro Plus (version 6.0). The result was presented as the ratio of the optical density of the phosphorylated target band to the total target or the *β*-actin band.

### 2.5. Quantitative Real-Time Polymerase Chain Reaction Analysis

Total RNA derived from each group was extracted with Trizol reagent. The purity and concentration of total RNA were measured by a nucleic acid/protein analyzer (Thermo, Rockford, IL, USA). Then 2 *μ*g of total RNA was reverse-transcribed using a PrimeScript RT reagent kit on a Mastercycler gradient polymerase chain reaction (PCR) apparatus (Eppendorf Company, Hamburg, Germany). The total reaction volume was 20 *μ*L. Then 2.0 *μ*L of cDNA was amplified in a 20 *μ*L PCR amplification reaction containing 0.4 *μ*L forward primer, 0.4 *μ*L reverse primer, 6.8 *μ*L ddH_2_O, 0.4 *μ*L ROX reference dye (50x), and 10.0 *μ*L SYBR Premix Ex Taq with an Applied Biosystems StepOne Real-Time PCR System (StepOne, Foster City, CA, USA). The whole process has three stages: Stage 1, 95°C for 30 s; Stage 2, 95°C for 5 s; and Stage 3, 60°C for 30 s. The method of 2^−ΔΔCT^ was used for data analysis. The primer sequences are given in Table 1.

### 2.6. Immunofluorescence Analysis

Cells were grown on glass microscope cover slides. After being modeled by insulin for 36 h, an intervention medium was added. Then the cells were fixed in 4% buffered formalin and permeabilized with 0.5% Triton X-100. Sections were incubated with either the GLUT-4 or the ER*α* antibody at dilution of 1 : 500 overnight at 4°C. After washing with PBS (pH = 7.4) three times, sections were incubated with the secondary antibody at dilution of 1 : 1000 for 1 h at room temperature. Then the sections were lightly counterstained with DAPI or WGA after washing. For immunofluorescence, slides were directly mounted in antifade mounting medium and visualized in a fluorescent microscopy (NIKON ECLIPSE CI or an Olympus Confocal Microscope model FV1000 at 800 × 600 pixel resolution). Image-Pro Plus 6.0 software was used for semiquantitative analysis of immunofluorescence.

### 2.7. Statistical Analysis

All results were presented as mean ± standard deviation (SD) and analyzed through SPSS 19.0 software. One-way analysis of variance (ANOVA) was used to determine the statistical significance. Based on whether data assumed equal variances or not, LSD or Dunnett's T3 test was used, respectively. *P* < 0.05 was considered statistically significant.

## 3. Results

### 3.1. Effects of DSG and 5-MOP on Expression of ER*α* in HepG2 Cells by Immunofluorescence

**Figure 2:**
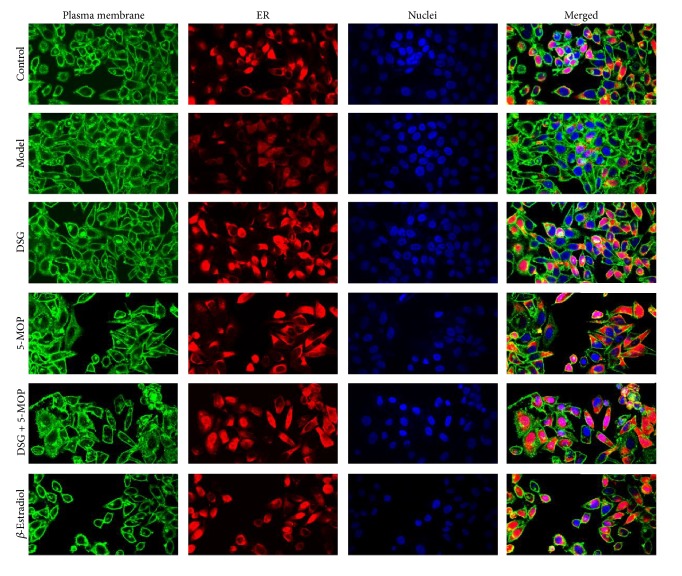
Effects of DSG and 5-MOP on expression of ER in HepG2 cells. HepG2 cells were stained for ER with Dylight 549 (red) and plasma membrane with WGA (green). Additionally, nuclei were stained with DAPI (blue). The merged images showed that DSG, 5-MOP, DSG + 5-MOP, and *β*-Estradiol led to an increase of the ER expression compared with the model group. Images were collected using an Olympus Confocal Microscope model FV1000 at 800 × 600-pixel resolution with a 60x objective lens.

Cells in the logarithmic growth phase were used for detecting the expression of ER*α*. In our study, double staining with ER*α* and plasma membrane revealed the presence of ER*α* in plasmalemma, cytoplasm, and nuclei by the use of confocal imaging. The merged images also showed that DSG, 5-MOP, DSG + 5-MOP, and *β*-Estradiol led to an increase of the ER expression compared with the model group (Figure 2).

### 3.2. DSG, 5-MOP, and *β*-Estradiol for Cell Viability

**Figure 3:**
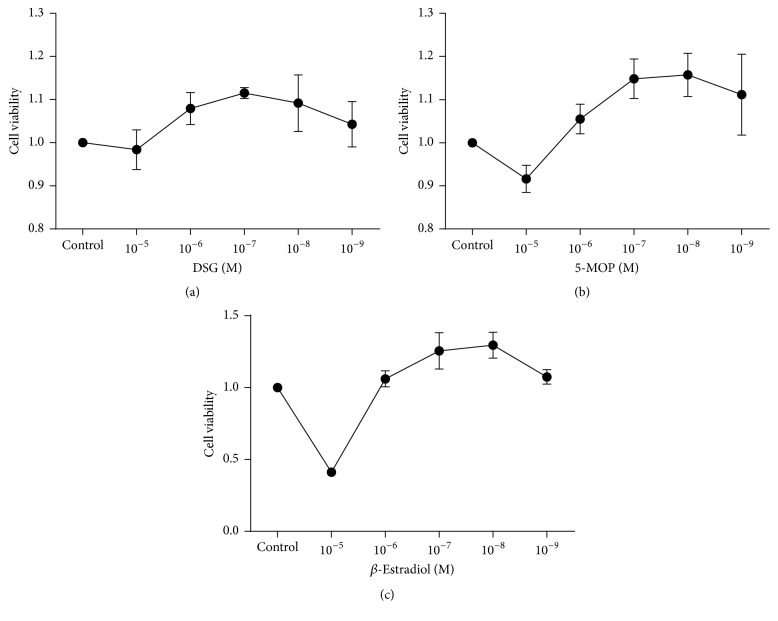
DSG, 5-MOP, and *β*-Estradiol for the cell viability. (a) DSG (10^−5 ^mol/L), (b) 5-MOP (10^−6 ^mol/L), and (c) *β*-Estradiol (10^−6 ^mol/L) showed negligible effect on cell viability.

Under the conditions of this study, the maximum nontoxic concentration was as follows: DSG (10^−5 ^mol/L), 5-MOP (10^−6 ^mol/L), and *β*-Estradiol (10^−6 ^mol/L) (Figure 3).

### 3.3. Effects of DSG and 5-MOP on Supernatant Glucose Content in HepG2 Cells

**Figure 4:**
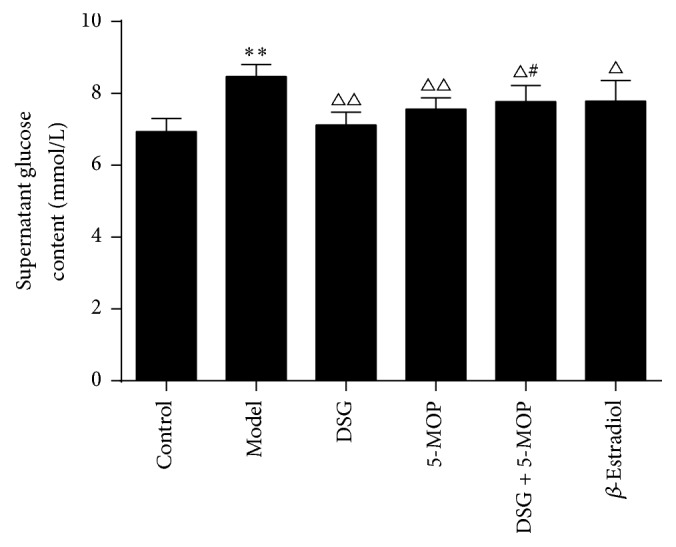
Effects of DSG and 5-MOP on supernatant glucose content in HepG2 cells. DSG, 5-MOP, DSG + 5-MOP, and *β*-Estradiol led to an increased glucose consumption. ^*∗∗*^ *P* < 0.01: significance from control group. ^△^ *P* < 0.05 and ^△△^ *P* < 0.01: significance from model group. ^#^ *P* < 0.05: significance from DSG-treated group. Each bar represents mean ± SD from three wells. Data were verified by viable cell counts.

Compared with the control group, culturing HepG2 cells in the presence of insulin (10^−6 ^mol/L) for 36 h caused a significant decrease in supernatant glucose consumption (*P* < 0.01) (Figure 4). However, treatment with DSG, 5-MOP, DSG + 5-MOP, and *β*-Estradiol led to an increased glucose consumption (*P* < 0.05, *P* < 0.01). The group treated with DSG + 5-MOP seemed to show a weaker effect on glucose consumption compared with DSG alone (*P* < 0.05). Data were verified by viable cell counts.

### 3.4. Effects of DSG and 5-MOP on Intracellular Glycogen Synthesis in HepG2 Cells

**Figure 5:**
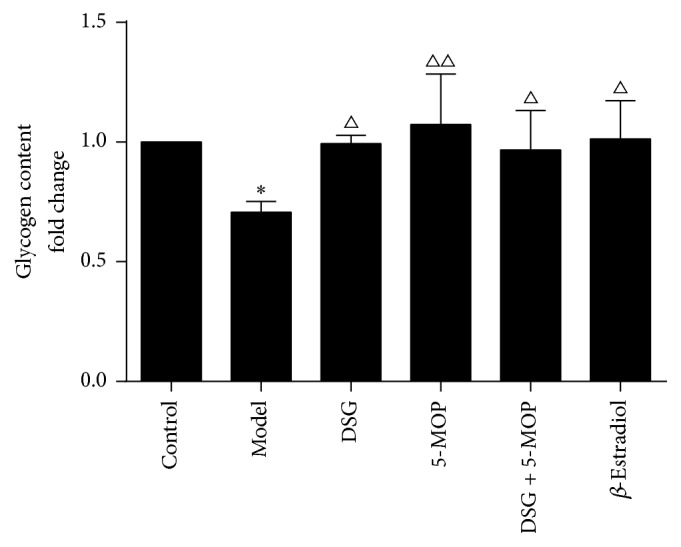
Effects of DSG and 5-MOP on intracellular glycogen synthesis in HepG2 cells. DSG, 5-MOP, DSG + 5-MOP, and *β*-Estradiol led to an increased intracellular glycogen content. ^*∗*^ *P* < 0.05: significance from control group. ^△^ *P* < 0.05 and ^△△^ *P* < 0.01: significance from model group. Each bar represents mean ± SD from three wells.

As shown in Figure 5, the situation of intracellular glycogen synthesis in the model group came to a low degree compared with control group (*P* < 0.05). Meanwhile, intracellular glycogen contents were remarkably increased in the DSG, 5-MOP, DSG + 5-MOP, and *β*-Estradiol groups (*P* < 0.05, *P* < 0.01). In this regard, the DSG + 5-MOP group did not present a more outstanding performance compared with the two herbal monomer groups.

### 3.5. Effects of DSG and 5-MOP on the Expression of Proteins in ER*α*/PI3K/Akt-Signaling Pathway

**Figure 6:**
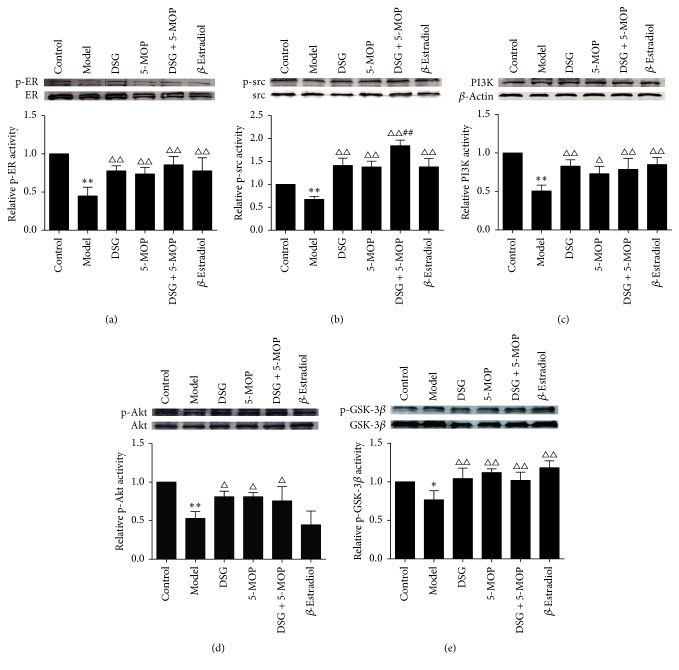
Effects of DSG and 5-MOP on the expression of proteins in PI3K/Akt pathways involving ER*α*. Representative protein levels for (a) p-ER (Tyr-537)/ER*α*, (b) p-Src (Tyr-416)/Src, (c) PI3Kp85/*β*-actin, (d) p-Akt (Ser473)/Akt, and (e) p-GSK-3*β* (Ser9)/GSK-3*β*. Each bar represents mean ± SD from three wells. ^*∗*^ *P* < 0.05 and ^*∗∗*^ *P* < 0.01: significance from control group. ^△^ *P* < 0.05 and ^△△^ *P* < 0.01: significance from model group. ^##^ *P* < 0.01: significance from DSG-treated group.

In order to explore the mechanism of DSG and 5-MOP on improving insulin resistance, we examined the protein expression on the PI3K/Akt pathway including p-Src (Tyr-416)/Src, PI3Kp85/*β*-actin, p-Akt (Ser473)/Akt, and p-GSK-3*β* (Ser9)/GSK-3*β*. We also detected ER*α*, which might be one upstream protein of this pathway and its phosphorylation site at Tyr-537. As shown in Figure 6, in our study, treatment with either DSG, 5-MOP, or their combination all exhibited a significant increase in the protein expression ratio mentioned above compared with the model group (*P* < 0.05, *P* < 0.01). No preferable effect appeared when the combination group was compared with the herbal monomer group except for their influence on p-Src/Src. The combination group (DSG + 5-MOP) led to more increase on p-Src/Src compared with the herbal monomer group (*P* < 0.01). Interestingly, *β*-Estradiol seemed to have no effects on the expression of p-Akt/Akt compared with the model group. This was different from its influence on other proteins, which showed a significant increase (*P* < 0.01).

### 3.6. Effects of DSG and 5-MOP on Gene Expression at the Transcriptional Level in ER*α*/PI3K/Akt-Signaling Pathway

**Figure 7:**
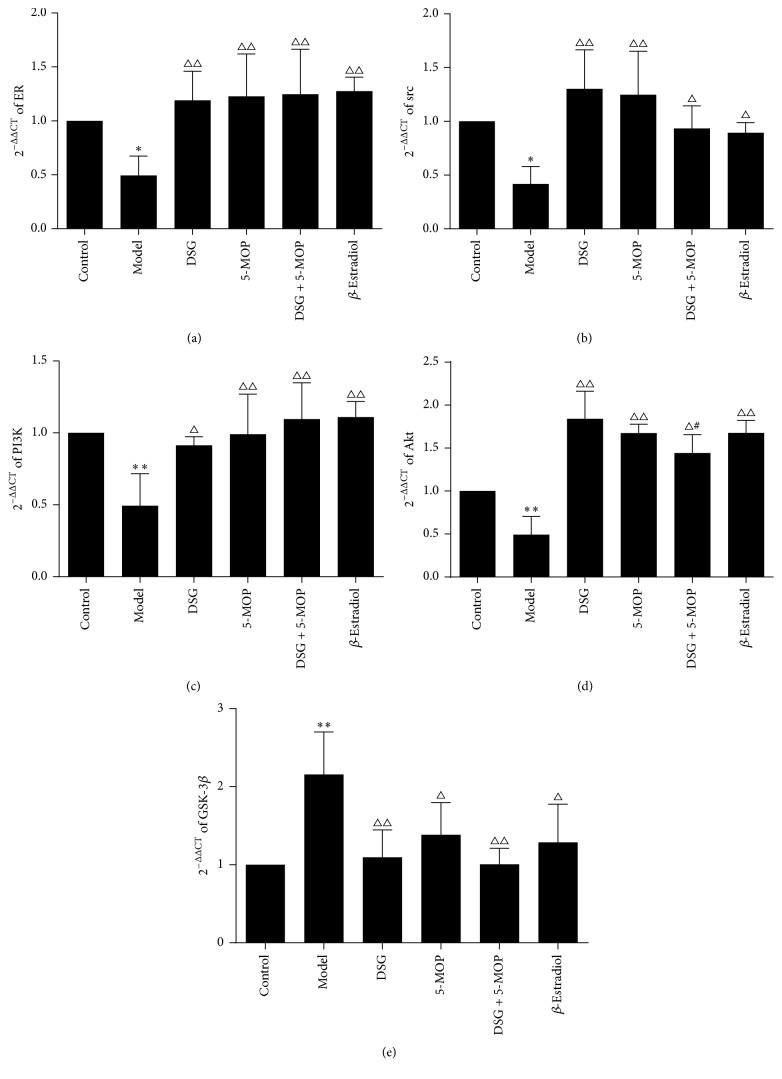
Effects of DSG and 5-MOP on gene expression at the transcriptional level in PI3K/Akt pathways involving ER*α*. Representative mRNA levels for (a) ER*α*, (b) Src, (c) PI3K p85, (d) Akt, and (e) GSK-3*β*. Each bar represents mean ± SD from three wells. ^∗^ *P* < 0.05 and ^∗∗^ *P* < 0.01: significance from control group. ^△^ *P* < 0.05 and ^△△^ *P* < 0.01: significance from model group. ^#^ *P* < 0.05: significance from DSG-treated group.

To detect the effects of DSG and 5-MOP on gene expression at the transcriptional level, we conducted RT-PCR. As shown in Figure 7, in our study, treatment with DSG, 5-MOP, their combination, and *β*-Estradiol all showed an increase in the gene transcription of ER*α*, Src, PI3Kp85, and Akt compared with the gene levels in the model group (*P* < 0.05, *P* < 0.01). On the contrary, negative regulation gene Gsk-3*β* displayed a decreasing expression after intervention (*P* < 0.05, *P* < 0.01). Also we found no difference between the combination group (DSG + 5-MOP) and their monomer group with regard to their promoting effects on gene transcription. Interestingly, we also found that the effect of DSG was better than the combination of DSG + 5-MOP on promoting the transcription of Akt (*P* < 0.05).

### 3.7. Effects of DSG and 5-MOP on Expression of GLUT-4 in HepG2 Cells

**Figure 8:**
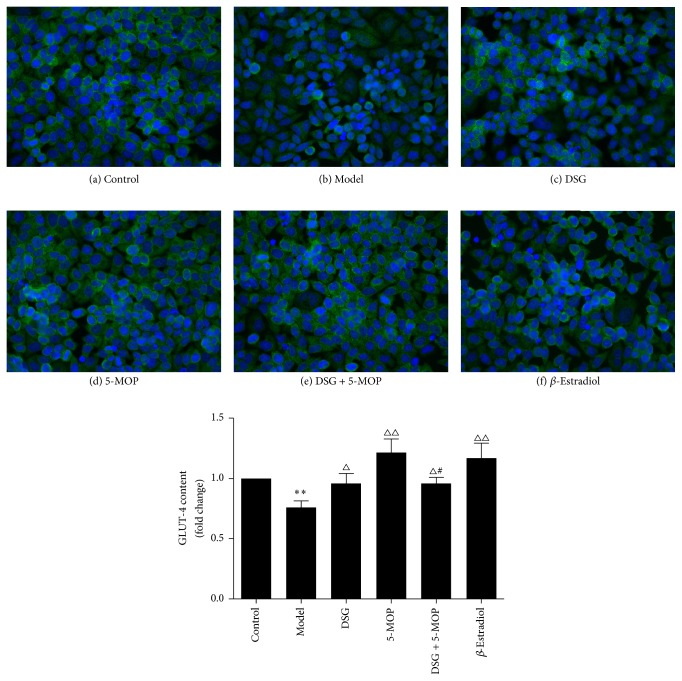
Effects of DSG and 5-MOP on expression of GLUT-4 (green) in HepG2 cells. Nuclei were also stained with DAPI (blue). Immunofluorescence showed that (c) DSG, (d) 5-MOP, (e) DSG + 5-MOP, and (f) *β*-Estradiol led to an increase of the GLUT-4 expression compared with the (b) model group. Each bar represents mean ± SD from three wells. ^*∗∗*^ *P* < 0.01: significance from control group. ^△^ *P* < 0.05 and ^△△^ *P* < 0.01: significance from model group. ^#^ *P* < 0.05: significance from 5-MOP-treated group.

To determine the effects of DSG and 5-MOP on the expression of GLUT-4, cells were evaluated by immunofluorescence after incubating with DSG, 5-MOP, DSG + 5-MOP, or *β*-Estradiol for 24 h. As shown in Figure 8, the expression of GLUT-4 had a significant increase in the treated groups compared with the model group (*P* < 0.05, *P* < 0.01) though no superior effect of the DSG + 5-MOP group appeared. On the contrary, the 5-MOP group showed a more prominent influence on increasing the content of GLUT-4 than did the DSG + 5-MOP group (*P* < 0.05).

## 4. Discussion

Diabetes mellitus is a chronic metabolic disorder caused by either impaired insulin secretion or a reduction in its biological effectiveness. T2DM is the predominant form of diabetes mellitus characterized by insulin resistance and it accounts for 90%–95% of the diabetic population [14]. In physiological conditions, the liver provides a main adjustment for glucostasis through synthesis and decomposition of glycogen, oxidative decomposition of glucose, and gluconeogenesis. When the liver works inappropriately, such as having a low sensitivity to insulin, it may lead to a fluctuation of blood glucose. Thus, improving the insulin resistance of the liver is an important direction for the treatment of T2DM.

In China, herbal medicines have been widely used as an alternative approach for treating T2DM [15]. Plants provide a vast treasure of natural products used as a primary source of medicine.* Trigonella foenum-graecum* and* Psoralea corylifolia* are two common drugs used for T2DM due to their yang-tonic effect. The main ingredients, DSG and 5-MOP, were chosen as the objectives in our study with the method of component compatibility [16]. In previous studies, DSG and 5-MOP showed multiple activities against glycometabolism disorder [17, 18]. Several studies attributed DSG's hypoglycemic effect to its regulation of metabolism-related enzymes [19, 20], its interaction with various target molecules, and the related signaling pathways [21, 22]. A recent study [23] explored the potential of DSG in the management of diabetes by ameliorating oxidative stress. An exploration of the mechanism of 5-MOP's effects on glucose control was also conducted, including its role in the inhibition of protein tyrosine phosphatase 1B [9] and oxidative stress [8]. However, the molecular mechanism concerning the effect of DSG and 5-MOP on glucose metabolism was far from being completely understood. Considering the estrogen-like effects of DSG and 5-MOP, we further explored the probable role of ER signaling in the regulation of glycometabolism in conjunction with the PI3K/Akt-signaling pathway, the direct signaling pathway that regulates glucose metabolism.

**Figure 9:**
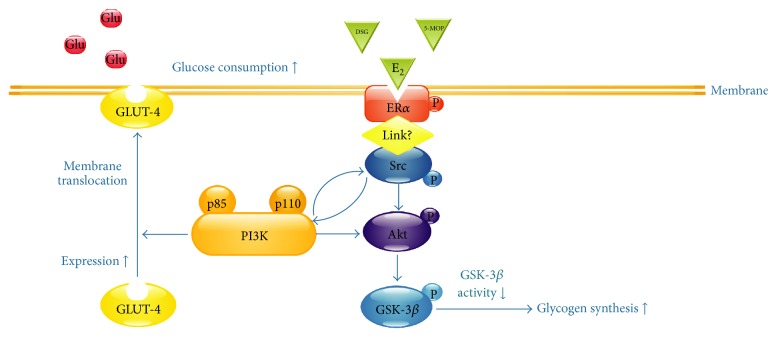
Assumed mechanism for *β*-Estradiol induced PI3K/AKT activation in HepG2 cells.

In recent years, the PI3K/Akt pathway has gained recognition for its role in metabolism regulation. Based on both in vivo and in vitro studies, PI3K is required for insulin-induced glucose uptake and the inhibition of glucose production [24, 25]. The PI3K regulatory isoform p85*α* gene has been reported in connection with increased risk of developing T2DM [26]. Many of the metabolic effects, including regulating glycometabolism, require activation of the PI3K downstream target Akt. Complete activation of Akt requires phosphorylation of ser473 at the C terminal. Constitutively active Akt induces translocation of the GLUT-4 to the plasma membrane to promote glucose uptake and also phosphorylate GSK-3*β* to increase glycogen synthesis [27, 28]. Src is an upstream protein of Akt. Phosphorylated Src at Tyr416 can activate Akt to complete the subsequent signal transduction [29]. A study conducted by Haynes et al. [30] indicated a complex of ER, c-Src, and PI3Kp85, which brought our attention to the ER-initiated membrane-localized steroid hormone receptor-signaling pathway. Therefore, we supposed the probable mechanism of *β*-Estradiol induced PI3K/Akt activation in HepG2 cell (Figure 9).

Our laboratory results showed that both DSG and 5-MOP could ameliorate insulin resistance as a result of accelerating glucose utility and intracellular glycogen synthesis. This phenomenon was accompanied by phosphorylation of ER*α*, Src, PI3Kp85, Akt, and Gsk-3*β*. Measurement of gene transcription was also conducted with a resultant increase in ER, Src, PI3Kp85, and Akt, but not for Gsk-3*β* after treatment by DSG or 5-MOP. Gsk-3*β* is a negative regulation molecule downstream of the PI3K/Akt pathway. After treatment, the gene expression of Gsk-3*β* decreased. Also, by means of immunofluorescence, we detected an increase of ER and GLUT-4, a sign of increased glucose utilization. However, the synergistic effect of DSG and 5-MOP on insulin resistance was not obvious in this study. DSG, a plant-derived steroid, serves as a precursor of various natural or synthetic steroidal hormones. It has a similar structure with estrogen, the primary female sex hormone, as discussed in our manuscript. As a mimic, we assumed that DSG might bind to the allosteric site of estrogen receptor immediately and reach a saturation state. In this case, the binding of DSG reduced the affinity of the estrogen receptor for 5-MOP, resulting in convulsions due to lessened inhibition of the estrogen mimic. Without DSG, 5-MOP would potentially exert an effect by activating the estrogen receptor. This may be the reason that no synergistic effect occurred when combining these two compounds. Nevertheless, the exact mechanism requires further investigation.

There are still some limitations of our work. In the present study, no direct evidence was given to certify the linkage between ER*α* and the PI3K pathway. Therefore further profound research is required.

In conclusion, our in vitro study suggests that DSG and 5-MOP may improve insulin resistance through an ER*α*-mediated PI3K/Akt activation pathway that implicates a novel therapeutic approach using this natural product in the treatment of metabolic diseases such as T2DM.

## Figures and Tables

**Table 1:** Real-time PCR primer sequences.

Gene	Forward (5′ → 3′)	Reverse (3′ → 5′)
*β*-Actin	CATGTACGTTGCTATCCAGGC	CTCCTTAATGTCACGCACGAT
ER-*α*	CATGAAGTGCAAGAACGTGGTG	AGGAAATGCGATGAAGTAGAGCC
Src	GAGCGGCTCCAGATTGTCAA	CTGGGGATGTAGCCTGTCTGT
PI3K p85	ACCACTACCGGAATGAATCTCT	GGGATGTGCGGGTATATTCTTC
Akt	CCTCAACAACTTCTCTGTGGCG	CACAGTCTGGATGGCGGTTG

## Abbreviations

HLBW: Hu-Lu-Ba-Wan
DSG: Diosgenin
5-MOP: 5-Methoxypsoralen
ER: Estrogen receptor
GLUT-4: Glucose transporter-4
T2DM: Type 2 diabetes mellitus.
